# Development and validation of a serial album on pregnancy for expectant mothers and healthcare professionals

**DOI:** 10.1590/0034-7167-2025-0284

**Published:** 2026-02-02

**Authors:** Leandro da Silva de Medeiros, Alice Guadagnini Leite, Marli Terezinha Stein Backes, Lurdes Lomba, Bruna Marta Kleinert Halberstadt, Dirce Stein Backes

**Affiliations:** I Universidade Franciscana. Santa Maria, Rio Grande do Sul, Brazil; II Universidade Federal de Santa Catarina. Florianópolis, Santa Catarina, Brazil; III Escola Superior de Enfermagem de Coimbra. Coimbra, Portugal

**Keywords:** Educational Technology, Health Education, Prenatal Care, Obstetric Nursing, Primary Health Care, Tecnología Educacional, Educación en Salud, Atención Prenatal, Enfermería Obstétrica, Atención Primaria de Salud

## Abstract

**Objectives:**

to describe the process of developing and validating a serial album on prenatal care aimed at pregnant women and health professionals.

**Methods:**

participatory action research that considered technological production guided by the following stages: identification of the need for change; theoretical and documentary survey; technological proposal; analysis by experts and validation by pregnant women. The analysis by experts considered the Content Validity Index.

**Results:**

the Content Validity Index obtained an overall score of 0.92, and the pregnant women performed the assessment with 100% agreement between items. The serial album on prenatal care proved to be effective in qualifying prenatal care, especially with regard to promoting autonomy, critical thinking, and strengthening the professional-user bond.

**Conclusions:**

the serial album developed was considered valid by pregnant women and health professionals and has the potential for replication in the context of primary health care, with a view to improving the quality of prenatal care.

## RESUMEN

**Objetivos:**

describir el proceso de desarrollo y validación de un álbum seriado sobre la atención prenatal dirigido a gestantes y profesionales de la salud.

**Métodos:**

investigación-acción participativa que contempló la producción tecnológica guiada por las etapas de: identificación de la necesidad de cambio; revisión teórica y documental; propuesta tecnológica; análisis por especialistas y validación por gestantes. El análisis por especialistas consideró el Índice de Validez de Contenido.

**Resultados:**

el Índice de Validez de Contenido obtuvo una puntuación global de 0,92; y las gestantes realizaron la evaluación con un 100% de concordancia entre los ítems. El álbum seriado sobre la atención prenatal se mostró eficaz para la mejora de la atención prenatal, especialmente en lo que respecta a la promoción de la autonomía, del pensamiento crítico y al fortalecimiento del vínculo profesional-usuaria.

**Consideraciones Finales:**

el álbum seriado desarrollado fue considerado válido por gestantes y profesionales de la salud y presenta potencial de replicabilidad en el contexto de la atención primaria de salud, con miras a la mejora de la atención prenatal.

## INTRODUCTION

Prenatal care in primary health care, using innovative tools that are accessible to both pregnant women and health professionals, is a forward-looking strategy for sustainable development^(1)^. The role of nurses in prenatal care is crucial, particularly with regard to the early identification of possible clinical complications and the establishment of a welcoming environment conducive to the development of educational practices that minimize adverse outcomes during labor, delivery, and the postpartum period^(2,3)^.

However, it is not enough to recognize the importance of prenatal care and the prospective role of nurses in promoting good obstetric practices capable of reducing unwanted outcomes^(4)^. The development of healthy and sustainable practices during pregnancy increasingly requires dialogical and participatory theoretical-methodological approaches, as well as digital and instructional technologies capable of supporting health professionals and that are widely accessible and attractive to users, in this case pregnant women^(5,6)^.

In Brazil, digital and instructional technologies are increasingly attractive and encouraged by funding agencies. Educational technologies are low-cost tools with wide reach for professionals and users and high effectiveness, among other advantages. The serial album, more specifically, is a visual educational tool capable of generating and democratizing knowledge from a collection of organized and systematized sheets (posters) that may contain maps, graphs, drawings, texts, and others. Among its advantages are: directing the construction of knowledge in a broad and participatory manner, enabling collegial decision-making, and intervening in the context in an effective and decisive manner^(7,8)^.

Recognizing the inductive potential of new and better practices in nursing and health through educational technologies, as well as the interactive role of nursing professionals in the context of primary health care, this study asks the following research question: can the serial album on prenatal care be considered a valid educational technology for use by pregnant women and health professionals?

## OBJECTIVES

To describe the process of developing and validating a serial album on prenatal care aimed at pregnant women and health professionals.

## METHODS

### Ethical aspects

The project was approved by the Research Ethics Committee of the Franciscan University. All research participants signed the Informed Consent Form (ICF). The ethical principles involving research with human beings were strictly observed.

### Study design, period, and stages

This is an action research study^(9)^ that sought to enable a movement to improve prenatal care, conducted between June 2024 and April 2025. In addition to the traditional stages of action research, this study included technological production, systematized by the following stages: 1. Identification of the need for change; 2. Theoretical and documentary survey; 3. Technological proposal; 4. Validation with health professionals; and 5. Validation with pregnant women. The EQUATOR network’s SQUIRE quality improvement studies were considered in the research process.

The first stage of the study, called identification of the need for change, was conducted in a systematic manner with the initial phases of action research. Pregnant women and postpartum women linked to a group of pregnant women from a Primary Care Center (PCC) located in a municipality in the central region of the state of Rio Grande do Sul (RS) participated. Data collection, carried out between October 2023 and April 2024, used individual interviews guided by a semi-structured script, covering sociodemographic issues and aspects related to the quality of prenatal care. The analysis of the data obtained allowed us to identify gaps in the care process, supporting the definition of priority content and themes for the construction of the serial album.

In the second stage of the study, a theoretical and documentary survey was conducted between June and August 2024, which supported the definition of the textual content of the serial album on prenatal care. This process involved a systematic search of official documents, with an emphasis on the guidelines recommended by the Ministry of Health, as well as clinical and care protocols in force at the municipal, state, and national levels. Normative and technical-scientific sources were analyzed, such as the pregnant woman’s handbook, the national guidelines for low-risk prenatal care, the technical manual for childbirth, birth, and postpartum care, as well as technical notes and manuals produced in the context of the maternal and child health care network. The selection of these materials was guided by criteria of timeliness, relevance to healthcare practice, and educational potential. This survey made it possible to identify the main thematic areas related to prenatal care, which guided the selection of content to be addressed in the album, ensuring its consistency with public policies.

In the third stage, technological proposal, the theoretical and documentary content was revisited to organize the editing and layout process, following criteria related to organization, language, layout and design, cultural sensitivity, and suitability for pregnant women and health professionals by a professional in the field of design, previously trained by the researchers and hired for this function. The illustrations used were created from original vectors from the Freepik website, carefully adapted manually in Adobe Illustrator software (version 2024). For the typography, Ainslie Sans was chosen, a contemporary and welcoming sans serif font that contributes to reinforcing the visual identity of the serial album with lightness, clarity, and accessibility.

The serial album proposal was submitted for consideration by students and health professionals who are members of a study group at a higher education institution. Fourteen health students, four resident nurses in obstetric nursing, and seven professionals working in maternity and primary health care from different municipalities in the central region of the state of Rio Grande do Sul participated in this prospective moment. The activity lasted two hours and took place remotely in August 2024, linked to the activities of a study group at a higher education institution. It should be noted that the participants had consolidated experience in teaching, research, and extension projects focused on maternal and child health, giving critical weight to the suggestions presented.

The contributions emerged in a qualitative manner and focused on different dimensions of the material, from the simplification and improvement of technical-popular language to aspects related to the graphic and didactic organization of the content. Also noteworthy was the recommendation to incorporate technical and technological products previously developed and validated by graduates of a professional graduate program, which were thematically related and complementary to the scope of the album. The suggestions were carefully analyzed by the researchers, considering their relevance, feasibility, and alignment with the objectives of the technological proposal. The relevant changes were integrated into the serial album proposal, which was then sent back to the designer for final graphic adjustments. The result of this collaborative and iterative process culminated in the development of the first version of the serial album, which was submitted for validation. For this stage, 15 printed copies of the serial album were initially produced, organized into 28 pages, intended exclusively for the evaluation and validation of the serial album.

In the fourth stage, the serial album developed was validated with health professionals who are specialists in the area of interest, working in management and care in the areas of women’s and children’s health. After analysis by the health professionals specializing in the area, the album was sent for final technical adjustments with the professional responsible for layout and graphic design. Finally, 150 copies were printed at a digital printing company, using 220g glossy A4 paper, colored on both sides, measuring 30x21cm, with a pink A4 spiral binding at the top.

The fifth and final stage of the study corresponded to validation with pregnant women linked to groups of pregnant women from two Primary Care Center located in a municipality in the central region of the state of Rio Grande do Sul. Initially, the serial album was presented collectively by the researchers. Then, each participant received a printed copy of the album and was invited to answer a form with 10 questions that sought to understand their opinions regarding the organization, language, visual aspect, and learning stimulus promoted by the serial album^(10)^.

### Study sample and period

Considering the diversity of references and the lack of standardized guidelines for defining the profile of specialists, the criteria for participation in the validation process, corresponding to the fourth stage of the study, were defined by the researchers. For content validation, judges with a degree in health; a lato sensu postgraduate degree in women’s and children’s health; a stricto sensu postgraduate degree in health; and at least one year of experience in prenatal care and/or childbirth were considered specialists.

Health professionals were invited to participate in the research at a regional scientific event, which brought together professionals working in the field of maternal and child health in September 2024 at a higher education institution. Those who met the criteria previously defined by the researchers and expressed interest in participating in the study provided their email addresses, signed the informed consent form, and received a printed copy of the serial album.

In addition, the invitation to participate was also extended by the 4^th^ Regional Health Coordination Office (RHCO) to the other 33 municipal health departments. The specialists who agreed to participate through this contact were asked to fill in identification data, such as email, health center, and municipality of practice. Then, the digital version of the serial album, the informed consent form, and guidelines on the evaluation procedure were sent. Participants were given a maximum of 30 days from the date the material was sent to return the duly completed instruments.

After confirming receipt of the material at the addresses provided and respecting the deadline for return, the final sample of the study consisted of 18 expert judges, 12 of whom received the serial album in digital format and 6 in printed format. All participants returned the assessment instruments and consent forms duly completed.

In the second stage of validation of the serial album, 12 pregnant women participated, a number described in the literature as the minimum number of informants^(11)^. The inclusion criteria were: being pregnant, regardless of gestational age; being 18 years of age or older; and being literate, with a minimum of four years of schooling. Pregnant women who had limitations to full participation in the serial album evaluation process were excluded.

### Data Collection and analysis of results

The evaluation of the serial album content by experts considered the criteria of objectives, structure, presentation, and relevance of the items^(12)^. For this stage, an inverted Likert scale with five rating levels was used: 1 = strongly disagree; 2 = disagree; 3 = neutral; 4 = agree; and 5 = strongly agree. Responses assigned to levels 1 and 2 required written justifications from the evaluators, and the corresponding items were reanalyzed by the researchers, who decided whether to keep, modify, or exclude the material. Responses with a score of “3”, expressing a neutral position, were not considered for the calculation of validation indices.

The evaluation carried out by the pregnant women considered aspects such as organization, writing style, appearance, and motivation to read the album^(10)^. For this purpose, dichotomous responses were used, including the opposite pairs “yes” or “no”, “easy to understand” or “difficult to understand”, “clear” or “confusing”, and “interesting” or “uninteresting”.

In the stage of analysis of the instrument by content experts, the Content Validity Index (CVI) was used^(13,14)^. The CVI measures the proportion of judges who agree on a particular aspect of the instrument and its items^(15)^. The index was calculated by dividing the number of items considered adequate by the experts, i.e., those rated 4 and 5, by the total number of items evaluated^(16)^. A CVI value equal to or greater than 0.80 (80%) was established as acceptable, both in the individual analysis of the items and in the overall evaluation of the serial album^(14)^.

Regarding the analysis of data from the evaluation by pregnant women, items that achieved a minimum percentage of 75% positive responses were considered validated. Responses such as “yes”, “easy to understand”, “clear”, and “interesting” were classified as positive^(17,18)^. The collected data were organized in Microsoft^®^ Office 365 spreadsheets, version 1812, and after coding and tabulation, analyzed using descriptive statistics. The recommendations provided by the experts were incorporated into the final version of the serial album.

## RESULTS

The following section presents the process of creating the prenatal serial album and the validation process for the prenatal serial album.

### Construction of the prenatal serial album

The first step in creating the serial album corresponded to the initial stages of action research conducted with pregnant women and new mothers, based on the identification of demands related to the qualification of practices in the field of Primary Health Care. Based on dialogues and agreements between professionals and users, there was a clear demand for educational technology focused on prenatal care. After several opportunities for dialogue and discussion, the serial album technology was chosen.

The subsequent stage involved theoretical and documentary research, which allowed the identification of priority topics to be addressed in the serial album. At this stage, content was selected based on scientific evidence and national and international regulatory documents, ensuring the inclusion of clear, objective information compatible with the target audience. Illustrations were incorporated to facilitate understanding of the educational messages.

The first version of the serial album consisted of 28 pages. Although the agreement rates obtained in the validation stage were high, the researchers incorporated suggestions from experts that were deemed relevant to improve the content and its visual presentation. Among the changes made, the following stand out: replacement of technical terms with commonly used terms; change of the term “birth plan” to “individual birth and delivery plan”; inclusion of guidelines on essential items for maternity care and on the right to visit the referral maternity hospital; detailing the organization of prenatal consultations according to gestational weeks; adding a page with content on vaccines; and diversifying the ethnic and racial backgrounds of the characters illustrated.

Based on the contributions of the experts, the final version of the serial album resulted in 30 pages. The material was systematized with organizational elements such as a cover, presentation of the album and authors, as well as pages with essential elements: first, second, and third trimesters of pregnancy; postpartum consultation; immediate care at home; a “Learn more” section with Quick Response codes (QR Codes) for educational videos; first aid; childcare consultations; and general guidelines. The content was organized, more specifically, into double pages: one aimed at health professionals and the other, on the back, aimed at pregnant women. A page presenting the study group responsible for developing the technology was also included, as well as the serial album’s International Standard Book Number (ISBN) registration number.

### Validation of the prenatal serial album

The serial album was validated by healthcare professionals and pregnant women. The validation stage with specialists involved 18 professionals, 16 women and two men, including eight nurses, one nutritionist, five doctors, and four dental surgeons. The average age of the participants was 34 years, with training ranging from four to twelve years; 16 worked in healthcare and three in management; two of the specialists had doctoral degrees. These professionals represented eight municipalities in the central region of the state of Rio Grande do Sul, respectively: Santa Maria, Tupanciretã, Dona Francisca, Restinga Seca, Faxinal do Soturno, São Francisco de Assis, Agudo, and Cacequi.

The overall CVI, calculated based on the average of all items, scored 0.92, considering the validated serial album, as shown in Chart 1 ^(14)^.

**Chart 1:** Content validation for the “Prenatal Serial Album”, Santa Maria, Rio Grande do Sul, Brazil, 2025, N = 18

Domains	Critérios de avaliação do álbum seriado	CVI
1. Objectives	1.1 The objectives are consistent with the health education needs of pregnant women and health professionals.	0.88
	1.2 The serial album is important for the prenatal care of pregnant women.	0.88
	1.3 The information in the serial album promotes reflection and behavioral changes related to prenatal care.	0.86
	1.4 The serial album can be used in academic and scientific settings.	0.88
	1.5 The album meets the objectives of health institutions focused on prenatal care.	0.88
2. Structure and presentation	2.1 The album is appropriate for pregnant women and health professionals.	0.88
	2.2 The text is clear and objective.	0.88
	2.3 The information is scientifically accurate.	0.88
	2.4 The material is appropriate for the sociocultural level of pregnant women.	0.86
	2.5 There is a logical sequence to the content presented.	1.00
	2.6 The information is structured coherently and spelled correctly.	0.88
	2.7 The wording of the album corresponds to the level of knowledge of pregnant women.	1.00
	2.8 The information on the cover, introduction, and acknowledgments is coherent.	0.88
	2.9 The size of the titles and subtitles is appropriate.	1.00
	2.10 The illustrations are expressive and sufficient.	0.88
	2.11 The printed (or digital) material is appropriate.	1.00
	2.12 The number of pages is appropriate.	0.88
3. Relevance	3.1 The topics cover key aspects of prenatal care that should be reinforced during prenatal consultations.	1.00
	3.2 The material allows for the application and adaptation of learning to different contexts and audiences.	0.88
	3.3 The album proposes the construction of knowledge based on the exchange between professionals and pregnant women.	1.00
	3.4 The album addresses issues that are fundamental to promoting the health of pregnant women.	0.88
	3.5 The album is suitable for use by any pregnant woman and health professional.	1.00

CVI - Content Validity Index.

The validation stage with pregnant women was carried out with 12 participants from groups of pregnant women from two Primary Care Centers (PCC) located in a municipality in the central region of the state of RS. It should be noted that the instrument was applied during the second part of the meetings, that is, after the topic previously planned for that activity had been addressed. Upon agreeing to participate, the pregnant women received the serial album and were free to handle it extensively for as long as necessary before responding to the assessment instrument. The average application time was approximately 25 minutes.

Regarding the profile of the pregnant women, the average age was 28 years; eight were multiparous and four were primiparous, with an average of eight years of schooling, all of whom were married. The instrument applied obtained 100% agreement in positive responses, evidencing the textual clarity and adequacy of the illustrations. Thus, the material was considered relevant for prenatal health education and promotion processes, especially in the context of Primary Health Care. It is also noteworthy for its contribution to promoting the autonomy of pregnant women, stimulating critical-reflective thinking regarding care decisions, and strengthening the bond between health professionals and users.

**Figure 1:**
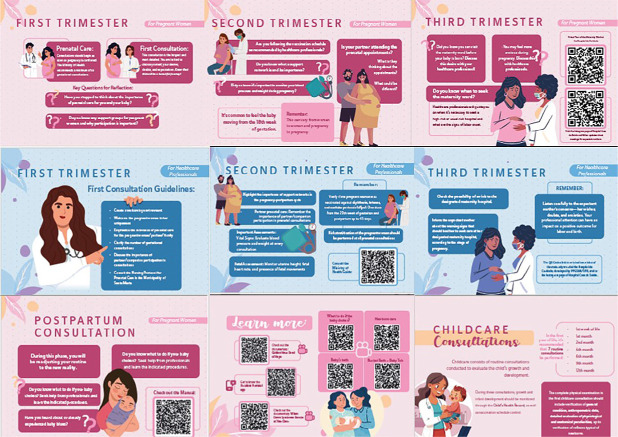
Pages from the “Prenatal Serial Album”, Santa Maria, Rio Grande do Sul, Brazil, 2025

During the validation process, some pregnant women reported doubts related to vaccines, individual birth plans, guided visits to the referral maternity hospital, and the Companion Law. These issues were clarified by the researchers at the time of application and highlighted as key points for improving prenatal care. The final version of the album consisted of 30 pages and was titled “Prenatal Serial Album” (Figure 1) and access link: https://pergamum.ufn.edu.br/pergamumweb/vinculos/0000c5/0000c57a.pdf.

## DISCUSSION

Although Brazil has made significant advances in maternal and child health, there are still important weaknesses in the development of educational technologies that support health promotion, especially in topics related to good practices in the pre-delivery, delivery, and post-delivery periods^(19)^. In this regard, the expanded action research project focused on improving prenatal care was fundamental in expanding interactive possibilities with health managers, professionals, and users and identifying specific demands, such as the development of this serial album, with a clear and illustrative configuration, as well as the potential to provide practical support to both pregnant women and health professionals.

During the development of this educational instructional technology, dialogical principles among the various participants were considered, as well as theoretical-practical approaches based on references capable of enabling the engagement and protagonism of professionals and users, attractive illustrative interfaces that induce new knowledge and practices, among others. The study demonstrated that educational technologies are tools that induce new knowledge and practices in health care, provided they are driven by horizontal and participatory approaches^(20)^.

The serial album, as an instructional technology to support education and health promotion, is a powerful resource for fostering disruptive processes and driving improvements in an interactive, associative, and participatory manner. It is not enough to identify demands and establish systematic health intervention plans. Beyond diagnosis, or identification of demands, it is necessary to design and prospect technologies that contribute to strengthening professional autonomy and the effective engagement of health users, in this case, pregnant women/families^(21,22)^.

Scholars have observed gaps in knowledge about prenatal care and deficiencies in skill domains among health professionals at various levels of the health system. Good prenatal coverage does not necessarily translate into the induction of good intra/postnatal practices, but rather into the ability to establish effective bonds^(23)^. Under this impetus and enhanced by attractive instructional tools, nurses have demonstrated interactive skills capable of fostering the protagonism of pregnant women, especially with regard to autonomous and responsible decision-making.

The validation index achieved in this study was higher than the minimum recommended value¹⁴. Studies highlight that the validation stage is fundamental for identifying convergences, points of misunderstanding, and the gap between the proposed content and its actual interpretation^(24,25)^. Thus, the evaluation carried out by health professionals and pregnant women showed that the serial album is consistent with their realities and needs, as demonstrated by the indices obtained. In summary, it should be noted that most of the specialists involved in the validation were nursing professionals, who acted as leaders and drivers of disruptive, innovative, and shared learning paths.

### Study limitations

The limitation of this study is associated with the impossibility of making theoretical and practical generalizations, given that the work, more specifically the serial album, was developed and validated based on a specific context in southern Brazil.

### Contributions to Nursing and Health Care

This study will contribute to the process of improving prenatal care, more specifically by providing a clear, illustrative, and evidence-based serial album. It is hoped that this instructional technology will contribute to improving prenatal care by expanding dialogic educational strategies for nursing professionals and empowering pregnant women.

## FINAL CONSIDERATIONS

The serial album developed was considered valid by pregnant women and health professionals and has the potential to be replicated in the context of primary health care, with a view to improving prenatal care. In addition to analyzing the items, the evaluators/specialists/pregnant women were able to suggest improvements and contribute effectively to the refinement of the final version of the serial album.

The serial album developed and validated can be considered, in short, an effective educational technology to support health professionals and pregnant women in understanding topics related to prenatal care and, thus, induce good practices that contribute to reducing maternal and infant morbidity and mortality rates and, ultimately, to achieving the Sustainable Development Goals.

## Data Availability

The research data are available within the article.
