# Supplementary material for: Development and validation of a serial album on pregnancy for expectant mothers and healthcare professionals
Source: Rev Bras Enferm. 2026 Feb 2;79:e20250284. doi: 10.1590/0034-7167-2025-0284 (PMC12872187; doi:10.1590/0034-7167-2025-0284)
Supplement: Translation

# Translation: Desenvolvimento e validação de álbum seriado sobre o pré-natal para gestantes e profissionais de saúde

**DOI:** 10.1590/0034-7167-2025-0284pt

**Authors:** Leandro da Silva de Medeiros, Alice Guadagnini Leite, Marli Terezinha Stein Backes, Lurdes Lomba, Bruna Marta Kleinert Halberstadt, Dirce Stein Backes

**Affiliations:** I Universidade Franciscana. Santa Maria, Rio Grande do Sul, Brasil; II Universidade Federal de Santa Catarina. Florianópolis, Santa Catarina, Brasil; III Escola Superior de Enfermagem de Coimbra. Coimbra, Portugal

**Keywords:** Tecnologia Educacional, Educação em Saúde, Cuidado Pré-Natal, Enfermagem Obstétrica, Atenção Primária à Saúde

## Abstract

**Objetivos:**

descrever o processo de desenvolvimento e validação de um álbum seriado sobre o pré-natal direcionado às gestantes e profissionais de saúde.

**Métodos:**

pesquisa-ação participante que considerou a produção tecnológica orientada pelas etapas: identificação da necessidade de mudança; levantamento teórico e documental; proposição tecnológica; análise por especialistas e validação por gestantes. A análise por especialistas considerou o Índice de Validade de Conteúdo.

**Resultados:**

o Índice de Validade de Conteúdo obteve escore global de 0,92; e as gestantes realizaram avaliação com 100% de concordância entre os itens. O álbum seriado sobre o pré-natal demonstrou-se eficaz à qualificação da atenção pré-natal, especialmente no que se refere à promoção da autonomia, do pensamento crítico e da ampliação do vínculo profissional-usuária.

**Considerações Finais:**

o álbum seriado desenvolvido foi considerado válido pelas gestantes e profissionais de saúde e com potencial de replicabilidade no contexto da atenção primária à saúde, com vistas à qualificação da assistência pré-natal.

## INTRODUÇÃO

A qualificação pré-natal em âmbito da Atenção Primária à Saúde, por meio de ferramentas inovadoras e acessíveis tanto às gestantes quanto aos profissionais de saúde, se constitui em estratégia prospectiva ao desenvolvimento sustentável^(1)^. A atuação do Enfermeiro no percurso pré-natal é um componente crucial, mais especificamente no que se refere à identificação precoce de possíveis complicações clínicas, bem como no estabelecimento de ambiência acolhedora e propícia ao desenvolvimento de práticas educativas de modo a minimizar desfechos desfavoráveis no parto, nascimento e pós-parto^(2,3)^.

Não basta, no entanto, reconhecer a relevância da qualificação pré-natal e o papel prospectivo do Enfermeiro na indução de boas práticas obstétricas capazes de reduzir desfechos indesejados^(4)^. O desenvolvimento de práticas saudáveis e sustentáveis no percurso gestacional demanda, crescentemente, abordagens teórico-metodológicas dialógicas e participativas, além de tecnologias digitais e instrucionais capazes de apoiar os profissionais de saúde e que sejam amplamente acessíveis e atraentes às usuárias, neste caso às gestantes^(5,6)^.

Em âmbito de Brasil, as tecnologias digitais e instrucionais são, cada vez mais, atrativas e estimuladas pelas agências financiadoras. As tecnologias educativas se constituem em ferramentas de baixo custo, de amplo alcance para profissionais e usuários, de alta efetividade, dentre outras vantagens. O álbum seriado, mais especificamente, é uma ferramenta visual educativa, capaz de gerar e democratizar o conhecimento, a partir de uma coleção de folhas (cartazes) organizadas e sistematizadas que podem conter mapas, gráficos, desenhos, textos e outros. Destacam-se, dentre as suas vantagens: direcionar a construção do conhecimento de forma ampla e participativa, possibilitar à tomada de decisões colegiadas, intervir no contexto de forma efetiva e resolutiva^(7,8)^.

Reconhecendo o potencial indutivo de novas e melhores práticas em Enfermagem e saúde, por meio das tecnologias educacionais, bem como o protagonismo interativo dos profissionais de Enfermagem no contexto da atenção primária à saúde, o presente estudo tem por questão pesquisa: o álbum seriado sobre o pré-natal pode ser considerado tecnologia educacional válida para ser utilizada por gestantes e profissionais de saúde?

## OBJETIVOS

Descrever o processo de desenvolvimento e validação de um álbum seriado sobre o pré-natal direcionado às gestantes e profissionais de saúde.

## MÉTODOS

### Aspectos éticos

O projeto foi aprovado pelo Comitê de Ética em Pesquisa da Universidade Franciscana. Todos os participantes da pesquisa assinaram o Termo de Consentimento Livre e Esclarecido (TCLE). Foram rigorosamente observados os princípios éticos que envolvem a pesquisa com seres humanos.

### Desenho, período e etapas do estudo

Trata-se de pesquisa-ação^(9)^ que intentou possibilitar um movimento de qualificação da atenção pré-natal, desenvolvida entre junho de 2024 a abril de 2025. Além das tradicionais etapas da pesquisa-ação foi acrescida, neste estudo, a produção tecnológica, sistematizada pelas seguintes etapas: 1. Identificação da necessidade de mudança; 2. Levantamento teórico e documental; 3. Proposição tecnológica; 4. Validação com profissionais da saúde; e 5. Validação com gestantes. Considerou-se no processo desta pesquisa o referencial de estudos de melhoria da qualidade-SQUIRE da rede EQUATOR.

A primeira etapa do estudo, denominada identificação da necessidade de mudança, foi conduzida de forma sistematizada com as fases iniciais da pesquisa-ação. Participaram gestantes e puérperas vinculadas a um grupo de gestantes de uma Unidade Básica de Saúde (UBS) situada em um município da região central do estado do Rio Grande do Sul (RS). Para a coleta de dados, realizada entre outubro de 2023 a abril de 2024, utilizaram-se entrevistas individuais guiadas por um roteiro semiestruturado, contemplando questões sociodemográficas e aspectos relacionados à qualificação da assistência pré-natal. A análise dos dados obtidos permitiu identificar lacunas no processo de cuidado, subsidiando a definição dos conteúdos e temas prioritários para a construção do álbum seriado.

Na segunda etapa do estudo, realizou-se o levantamento teórico e documental, entre junho e agosto de 2024, que subsidiou a definição dos conteúdos textuais do álbum seriado sobre o pré-natal. Esse processo envolveu uma busca sistemática em documentos oficiais, com ênfase nas diretrizes preconizadas pelo Ministério da Saúde, bem como em protocolos clínicos e assistenciais vigentes em esferas municipal, estadual e nacional. Foram analisadas fontes normativas e técnico-científicas, como a caderneta da gestante, as diretrizes nacionais de assistência ao pré-natal de baixo risco, o manual técnico de atenção ao parto, nascimento e puerpério, além de notas técnicas e manuais produzidos no contexto da rede de atenção à saúde materno infantil. A seleção desses materiais foi orientada pelos critérios de atualidade, relevância para a prática assistencial e potencial educativo. Esse levantamento permitiu identificar os principais eixos temáticos relacionados à assistência pré-natal, os quais nortearam a seleção dos conteúdos a serem abordados no álbum, assegurando sua consonância com as políticas públicas.

Na terceira etapa, proposição tecnológica, retomou-se o conteúdo teórico e documental para organizar o processo de edição e diagramação, obedecendo a critérios relacionados a organização, linguagem, layout e design, sensibilidade cultural e adequação às gestantes e profissionais da saúde por profissional da área de designer, previamente instrumentada pelos pesquisadores e contratada para esta função. As ilustrações utilizadas foram elaboradas a partir de vetores originais do site Freepik, cuidadosamente adaptados manualmente no *software Adobe Illustrator* (versão 2024). Para a tipografia, optou-se pela *Ainslie Sans*, uma fonte sem serifa de estilo contemporâneo e acolhedor, que contribui para reforçar a identidade visual do álbum seriado com leveza, clareza e acessibilidade.

A proposição do álbum seriado foi submetida à apreciação de estudantes e profissionais da saúde integrantes de um grupo de estudos de uma instituição de Ensino Superior. Participaram desse momento prospectivo 14 estudantes da área da saúde, 4 enfermeiras residentes em Enfermagem Obstétrica e 7 profissionais com atuação em maternidade e na Atenção Primária à Saúde, provenientes de distintos municípios da região central do estado do RS. A atividade teve a duração de duas horas e ocorreu de forma remota, no mês de agosto de 2024, vinculada às atividades de um grupo de estudos de uma Instituição de Ensino Superior. Ressalta-se que os participantes detinham experiência consolidada em projetos de ensino, pesquisa e extensão voltados à saúde materno infantil, conferindo densidade crítica às sugestões apresentadas.

As contribuições emergiram de maneira qualitativa e incidiram sobre diferentes dimensões do material, desde a simplificação e aprimoramento da linguagem técnico-popular, até aspectos relacionados à organização gráfica e didática do conteúdo. Destacou-se, ainda, a recomendação para a incorporação de produtos técnicos e tecnológicos previamente desenvolvidos e validados por egressos de um programa de pós-graduação profissional, os quais apresentavam afinidade temática e complementaridade ao escopo do álbum. As sugestões foram criteriosamente analisadas pelos pesquisadores, considerando-se sua relevância, viabilidade e alinhamento com os objetivos da proposta tecnológica. As alterações pertinentes foram integradas à proposição do álbum seriado que foi, em seguida, reenviada à designer para os ajustes gráficos finais. O resultado desse processo colaborativo e iterativo culminou na elaboração da primeira versão do álbum seriado, que foi submetida ao processo de validação. Para essa etapa, foram inicialmente produzidos 15 exemplares impressos do álbum seriado, organizados em 28 páginas, destinados exclusivamente à avaliação e validação do álbum seriado.

Na quarta etapa, realizou-se a validação do álbum seriado desenvolvido com profissionais da saúde especialistas da área de interesse, com atuação na gestão e assistência em áreas da saúde da mulher e da criança. Após análise dos profissionais da saúde especialistas, o álbum foi encaminhado para ajustes técnicos finais com a profissional responsável pela diagramação e design gráfico. Por fim, foram impressos 150 exemplares em gráfica de impressão digital, utilizando papel Couchê brilhoso A4 220g, colorido frente e verso, medindo 30x21cm, com espiral A4, cor rosa, na parte superior.

A quinta e última etapa do estudo correspondeu à validação com gestantes vinculadas a grupos de gestantes de duas Unidades Básicas de Saúde localizadas em um município da região central do estado do RS. Inicialmente, o álbum seriado foi apresentado de forma coletiva pelos pesquisadores. Em seguida, cada participante recebeu um exemplar impresso do álbum e foi convidada a responder um formulário com 10 questões que buscavam compreender suas opiniões quanto à organização, linguagem, aspecto visual e estímulo à aprendizagem promovido pelo álbum seriado^(10)^.

### Amostra e período do estudo

Considerando a diversidade de referenciais e a inexistência de diretrizes padronizadas para definição do perfil de especialistas, os critérios para participação no processo de validação, correspondente à quarta etapa do estudo, foram definidos pelos pesquisadores. Para validação de conteúdo, foram considerados especialistas os juízes que tivessem graduação na área da saúde; pós-graduação *lato-sensu* na área da saúde mulher e da criança; pós-graduação *stricto sensu* na área da saúde; e experiência mínima de um ano na assistência ao pré-natal e/ou parto.

Os profissionais da saúde especialistas foram convidados a participar da pesquisa por ocasião de um evento científico regional, o qual reuniu profissionais com atuação na área da saúde materno-infantil, em setembro de 2024, em uma instituição de ensino superior. Aqueles que atendiam aos critérios previamente definidos pelos pesquisadores e manifestaram interesse em participar do estudo forneceram seus endereços de *e-mail*, assinaram o TCLE e receberam uma cópia impressa do álbum seriado.

Complementarmente, o convite para participação também foi realizado pela 4ª Coordenadoria Regional de Saúde (CRS) para as demais 33 secretarias municipais de saúde. Os especialistas que aceitaram participar por meio desse contato, foi solicitado o preenchimento de dados de identificação, como *e-mail*, unidade de saúde e município de atuação. Em seguida, foi encaminhada a versão digital do álbum seriado, o TCLE e orientações sobre o procedimento de avaliação. Concedeu-se aos participantes o prazo máximo de 30 dias, contados a partir do envio do material, para devolução dos instrumentos devidamente preenchidos.

Após a confirmação do recebimento do material nos endereços informados e respeitado o prazo estabelecido para devolução, compuseram a amostra final do estudo 18 juízes especialistas, dos quais 12 receberam o álbum seriado em versão digital e 6 em versão impressa. Todos os participantes retornaram os instrumentos avaliativos e os termos de consentimento devidamente preenchidos.

Na segunda etapa de validação do álbum seriado, participaram 12 gestantes, quantitativo descrito na literatura como o mínimo de informantes^(11)^. Foram constituídos os critérios de inclusão: estar gestante, independentemente da idade gestacional; ter idade igual ou superior a 18 anos; e ser alfabetizada, com escolaridade mínima de quatro anos de estudo. Foram excluídas gestantes que apresentassem limitações para a participação plena no processo de avaliação do álbum seriado.

### Coleta e análise dos resultados

A avaliação do conteúdo do álbum seriado pelos especialistas contemplou os critérios de objetivos, estrutura, apresentação e relevância dos itens^(12)^. Para essa etapa, utilizou-se a escala tipo *Likert* invertida com cinco níveis de valoração:1 = discordo totalmente; 2 = discordo; 3 = neutro; 4 = concordo; e 5 = concordo totalmente. As respostas atribuídas aos níveis 1 e 2 demandaram justificativas escritas por parte dos avaliadores, sendo os itens correspondentes reanalisados pelas pesquisadoras, que deliberaram sobre sua permanência, modificação ou exclusão do material. Já as respostas com pontuação “3”, por expressarem posicionamento neutro, não foram consideradas para o cálculo dos índices de validação.

A avaliação realizada pelas gestantes considerou aspectos quanto à organização, o estilo de escrita, a aparência e a motivação para a leitura do álbum^(10)^. Para esse fim, foram utilizadas respostas dicotômicas, incluindo os pares opostos “sim” ou “não”, “fáceis de entender” ou “difíceis de entender”, “claro” ou “confuso” e “interessante” ou “desinteressante”.

Na etapa de análise do instrumento pelos especialistas em conteúdo, utilizou-se o Índice de Validade de Conteúdo (IVC)^(13,14)^. O IVC mede a proporção dos juízes que estão em concordância sobre determinado aspecto do instrumento e de seus itens^(15)^. O cálculo do índice foi realizado dividindo-se o número de itens considerados adequados pelos especialistas, isto é, aqueles avaliados com escores 4 e 5, pelo total de itens avaliados^(16)^. Foi estabelecido como aceitável um valor de IVC igual ou superior a 0,80 (80%), tanto na análise individual dos itens quanto na avaliação global da cartilha^(14)^.

Quanto à análise dos dados provenientes da avaliação pelas gestantes, foram considerados validados os itens que alcançaram um percentual mínimo de 75% de respostas positivas. Classificaram-se como positivas as respostas “sim”, “fáceis de entender”, “claro” e “interessante”^(17,18)^. Os dados coletados foram organizados em planilhas do *Microsoft* ^®^ *Office* 365, versão 1812, e após processo de codificação e tabulação, analisados por meio de estatística descritiva. As recomendações fornecidas pelos especialistas foram incorporadas na versão final do álbum seriado.

## RESULTADOS

Apresenta-se, na sequência, o percurso de construção do álbum seriado pré-natal e o processo de validação do álbum seriado pré-natal.

### Construção do álbum seriado pré-natal

O primeiro passo à construção do álbum seriado correspondeu às etapas iniciais da pesquisa-ação desenvolvida com gestantes e puérperas, a partir da identificação de demandas relacionadas à qualificação das práticas em âmbito da Atenção Primária à Saúde. Denotou-se, com base nos diálogos e acordos entre profissionais e usuários a demanda por uma tecnologia educacional voltada ao pré-natal. Após diversos espaços de interlocução e discussão chegou-se à tecnologia do álbum seriado.

A etapa subsequente envolveu o levantamento teórico e documental, que permitiu identificar os tópicos prioritários a serem abordados no álbum seriado. Nessa fase, selecionou-se conteúdos com base em evidências científicas e documentos normativos nacionais e internacionais, garantindo a inserção de informações claras, objetivas e compatíveis com o público-alvo. Ilustrações foram incorporadas com o objetivo de facilitar a compreensão das mensagens educativas.

A primeira versão do álbum seriado foi composta por 28 páginas. Embora os índices de concordância, obtidos na etapa de validação, tenham sido elevados, os pesquisadores incorporaram sugestões dos especialistas que foram julgadas pertinentes para aprimorar o conteúdo e sua apresentação visual. Dentre as modificações realizadas, destacam-se: substituição de termos técnicos por termos de uso popular; alteração do termo “plano de parto” por “plano individual de parto e nascimento”; inclusão de orientações sobre itens essenciais com os cuidados prestados na maternidade e sobre o direito à visita na maternidade de referência; detalhamento da organização das consultas pré-natais conforme as semanas gestacionais; adição de uma página com conteúdo sobre vacinas; e diversificação étnico-racial dos personagens ilustrados.

Com base nas contribuições dos especialistas, a versão final do álbum seriado resultou em 30 páginas. O material foi sistematizado com elementos organizacionais como capa, apresentação do álbum e dos autores, além de páginas com elementos essenciais: primeiro, segundo e terceiro trimestres gestacionais; consulta puerperal; cuidados imediatos no domicílio; seção “Saiba mais” com *Quick Response code* (QR Code) de vídeos educativos; primeiros socorros; consultas de puericultura; e orientações gerais. O conteúdo foi organizado, mais especificamente, em páginas duplas: uma direcionada aos profissionais de saúde e a outra, no verso, direcionada às gestantes. Também foi incluída uma página de apresentação do grupo de estudos responsável pela elaboração da tecnologia, bem como o número de registro do álbum seriado junto ao *International Standard Book Number* (ISBN).

### Validação do álbum seriado pré-natal

A validação do álbum seriado foi realizada por profissionais de saúde e com gestantes. A etapa de validação com os especialistas contou com a participação de 18 profissionais, sendo 16 mulheres e dois homens, distribuídos entre oito enfermeiros, uma nutricionista, cinco médicos e quatro cirurgiões-dentistas. A média de idade dos participantes foi de 34 anos, com tempo de formação variando entre quatro e doze anos; 16 atuavam na área assistencial e três na gestão; dois dos especialistas possuíam título de doutorado. Esses profissionais representaram oito municípios da região central do estado do RS, respectivamente: Santa Maria, Tupanciretã, Dona Francisca, Restinga Seca, Faxinal do Soturno, São Francisco de Assis, Agudo e Cacequi.

O IVC global, calculado com base na média de todos os itens, obteve pontuação de 0,92, considerando o álbum seriado validado, conforme apresentado na Quadro 1 ^(14)^.

**Quadro 1:** Validação de conteúdo “Álbum seriado Pré-natal”, Santa Maria, Rio Grande do Sul, Brasil, 2025, N = 18

Domínios	Critérios de avaliação do álbum seriado	IVC
1. Objetivos	1.1 Os objetivos são coerentes com as necessidades de educação em saúde de gestantes e profissionais da saúde.	0,88
	1.2 O álbum seriado é importante para o cuidado pré-natal das gestantes.	0,88
	1.3 As informações do álbum seriado promovem reflexões e mudanças de comportamento relacionadas ao pré-natal.	0,86
	1.4 O álbum seriado pode ser utilizado em espaços acadêmicos e científicos.	0,88
	1.5 O álbum atende aos objetivos de instituições de saúde voltadas ao cuidado pré-natal.	0,88
2. Estrutura e apresentação	2.1 O álbum é apropriado para gestantes e profissionais da saúde.	0,88
	2.2 O texto está claro e objetivo.	0,88
	2.3 As informações estão cientificamente corretas.	0,88
	2.4 O material está apropriado ao nível sociocultural das gestantes.	0,86
	2.5 Existe uma sequência lógica do conteúdo apresentado.	1,00
	2.6 As informações estão estruturadas com coerência e ortografia adequada.	0,88
	2.7 A redação do álbum corresponde ao nível de conhecimento das gestantes.	1,00
	2.8 As informações da capa, apresentação e agradecimentos são coerentes.	0,88
	2.9 O tamanho dos títulos e subtítulos está adequado.	1,00
	2.10 As ilustrações são expressivas e suficientes.	0,88
	2.11 O material impresso (ou digital) está adequado.	1,00
	2.12 O número de páginas está adequado.	0,88
3. Relevância	3.1 Os temas abordam postos-chaves do cuidado pré-natal que devem ser reforçados na consulta pré-natal.	1,00
	3.2 O material permite aplicação e adaptação do aprendizado a diferentes contextos e públicos.	0,88
	3.3 O álbum propõe a construção do conhecimento com base na troca entre profissional e gestante.	1,00
	3.4 O álbum aborda assuntos fundamentais à promoção da saúde da gestante.	0,88
	3.5 O álbum está adequado para ser utilizado por qualquer gestante e profissional da saúde.	1,00

*IVC - Índice de Validade de Conteúdo*.

A etapa de validação com gestantes foi realizada com 12 participantes de grupos de gestantes de duas Unidades Básicas de Saúde (UBS) situadas em um município da região central do estado do RS. Ressalta-se que a aplicação do instrumento ocorreu no segundo momento dos encontros, ou seja, após a abordagem do tema previamente planejado para aquela atividade. Mediante aceite para participação, as gestantes receberam o álbum seriado e tiveram a liberdade para manuseá-lo amplamente pelo tempo necessário, antes de responderem o instrumento de avaliação. O tempo médio de aplicação foi de aproximadamente 25 minutos.

Em relação ao perfil das gestantes, a média de idade foi de 28 anos; oito eram multíparas e quatro primíparas, com escolaridade média de oito anos, sendo todas casadas. O instrumento aplicado obteve 100% de concordância em respostas positivas, evidenciando a clareza textual e a adequação das ilustrações. Dessa forma, o material foi considerado pertinente para os processos de educação e promoção da saúde no pré-natal, sobretudo, em âmbito da Atenção Primária à Saúde. Destaca-se, ainda, sua contribuição à promoção da autonomia das gestantes, o estímulo ao pensamento crítico-reflexivo face às decisões relativas ao cuidado, além do fortalecimento do vínculo entre profissionais de saúde e usuárias.

Durante o processo de validação, algumas gestantes relataram dúvidas relacionadas às vacinas, ao plano individual de parto e nascimento, à visita guiada à maternidade de referência e à Lei do Acompanhante. Essas questões foram esclarecidas pelos pesquisadores no momento da aplicação e destacadas como pontos fundamentais para o aprimoramento da assistência pré-natal. A versão final do álbum foi constituída por 30 páginas e intitulado “Álbum Seriado Pré-Natal” (Figura 1) e link de acesso: https://pergamum.ufn.edu.br/pergamumweb/vinculos/0000c5/0000c57a.pdf.

**Figura 1:** Páginas do “Álbum Seriado Pré-natal”, Santa Maria, Rio Grande do Sul, Brasil, 2025

## DISCUSSÃO

Embora o Brasil tenha alcançado avanços significativos na saúde materno-infantil, ainda persistem fragilidades importantes no desenvolvimento de tecnologias educacionais que ofereçam suporte à promoção da saúde, especialmente em temas relacionados às boas práticas no percurso pré-parto, parto e pós-parto^(19)^. Nessa direção, o projeto ampliado de pesquisa-ação centrado na qualificação da atenção pré-natal foi fundamental para ampliar possibilidades interativas com gestores, profissionais e usuários de saúde e identificar demandas específicas, a exemplo do desenvolvimento deste álbum seriado, com configuração clara e ilustrativa, além de potencial para fornecer suporte prático tanto às gestantes quanto aos profissionais de saúde.

Considerou-se, no percurso de desenvolvimento dessa tecnologia instrucional educativa, princípios dialógicos entre os diversos participantes, abordagens teórico-práticas embasadas em referenciais capazes de possibilitar engajamento e o protagonismo de profissionais e usuários, interfaces ilustrativas atraentes e indutoras de novos saberes e práticas, dentre outros. Estudo demonstrou, Sob esse impulso, que as tecnologias educacionais se constituem em ferramentas indutivas de novos saberes e práticas no cuidado em saúde, desde que impulsionadas com abordagens horizontalizadas e participativas^(20)^.

O álbum seriado enquanto tecnologia instrucional de apoio à educação e promoção da saúde, se constitui em recurso potente para fomentar processos disruptivos e conduzir melhorias de forma interativa, associativa e participativa. Não basta identificar demandas e estabelecer planos sistemáticos de intervenção em saúde. Para além do diagnóstico, ou identificação de demandas, é preciso conceber e prospectar tecnologias que contribuam para o fortalecimento da autonomia profissional e o engajamento efetivo dos usuários de saúde, neste caso, as gestantes/famílias^(21,22)^.

Lacunas de conhecimento sobre o pré-natal e deficiências em domínios de habilidades entre profissionais de saúde em vários níveis do sistema de saúde foram observadas por estudiosos. Uma boa cobertura pré-natal não se traduz necessariamente na indução de boas práticas intra/pós-natais, mas na habilidade de estabelecer vínculos efetivos^(23)^. Sob esse impulso e potencializadas por ferramentas instrucionais atrativas, o profissional Enfermeiro tem demostrado habilidades interativas capazes de fomentar o protagonismo das gestantes, especialmente no que se refere à tomada de decisões autônomas e responsáveis.

O índice de validação alcançado neste estudo foi superior ao valor mínimo recomendado¹⁴. Estudos destacam que a etapa de validação é fundamental para identificar convergências, pontos de incompreensão e o distanciamento entre o conteúdo proposto e sua real interpretação^(24,25)^. Assim, a avaliação realizada por profissionais de saúde e gestantes evidenciou que o álbum seriado está consonante com suas realidades e necessidades, conforme demonstrado pelos índices obtidos. Destaca-se, em suma, que a maioria dos especialistas envolvidos na validação era composta por profissionais de Enfermagem, o qual figurou, nessa engrenagem, como líder agregador e condutor de percursos disruptivos, inovadores e de aprendizados compartilhados.

### Limitações do estudo

A limitação deste estudo está associada a impossibilidade de realizar generalizações teóricas e práticas, tendo em vista que o trabalho, mais especificamente o álbum seriado, foi desenvolvido e validado a partir de um contexto específico, no sul do Brasil.

### Contribuições para a Área de Enfermagem e Saúde

O presente estudo contribuirá no processo de qualificação do pré-natal, mais especificamente pela disponibilização de um álbum seriado claro, ilustrativo e baseado em evidências científicas. Espera-se, que essa tecnologia instrucional contribua à qualificação da assistência pré-natal pela ampliação de estratégias educativas dialógicas para os profissionais de Enfermagem e de empoderamento às gestantes.

## CONSIDERAÇÕES FINAIS

O álbum seriado desenvolvido foi considerado válido pelas gestantes e profissionais de saúde e com potencial de replicabilidade no contexto da atenção primária à saúde, com vistas à qualificação da assistência pré-natal. Além da análise dos itens, os avaliadores/especialistas/gestantes puderam sugerir melhorias e contribuir, efetivamente, para o aprimoramento da versão final do álbum seriado.

O álbum seriado desenvolvido e validado pode ser considerado, em suma, em uma tecnologia educativa eficaz para apoiar profissionais de saúde e gestantes na compreensão de temas relacionados ao pré-natal e, dessa forma, induzir boas práticas que contribuam para a redução das taxas de morbimortalidade materna e infantil e, em última análise, para o alcance dos Objetivos de Desenvolvimento Sustentável.

## Data Availability

Os dados da pesquisa estão disponíveis no corpo do artigo.
